# A three-feature prediction model for metastasis-free survival after surgery of localized clear cell renal cell carcinoma

**DOI:** 10.1038/s41598-021-88177-9

**Published:** 2021-04-21

**Authors:** Kalle E. Mattila, Teemu D. Laajala, Sara V. Tornberg, Tuomas P. Kilpeläinen, Paula Vainio, Otto Ettala, Peter J. Boström, Harry Nisen, Laura L. Elo, Panu M. Jaakkola

**Affiliations:** 1 Department of Oncology and Radiotherapy, Fican West Cancer Centre, University of Turku and Turku University Hospital, Hämeentie 11, Post Box 52, 20521 Turku, Finland; 2 Biomathematics Research Group, Fican West Cancer Centre, University of Turku, Turku, Finland; 3 Turku Bioscience Centre, University of Turku and Åbo Akademi University, Turku, Finland; 4 Department of Urology, University of Helsinki and Helsinki University Hospital, Helsinki, Finland; 5 Department of Pathology, University of Turku and Turku University Hospital, Turku, Finland; 6 Department of Urology, University of Turku and Turku University Hospital, Turku, Finland

**Keywords:** Oncology, Risk factors, Urology, Mathematics and computing

## Abstract

After surgery of localized renal cell carcinoma, over 20% of the patients will develop distant metastases. Our aim was to develop an easy-to-use prognostic model for predicting metastasis-free survival after radical or partial nephrectomy of localized clear cell RCC. Model training was performed on 196 patients. Right-censored metastasis-free survival was analysed using LASSO-regularized Cox regression, which identified three key prediction features. The model was validated in an external cohort of 714 patients. 55 (28%) and 134 (19%) patients developed distant metastases during the median postoperative follow-up of 6.3 years (interquartile range 3.4–8.6) and 5.4 years (4.0–7.6) in the training and validation cohort, respectively. Patients were stratified into clinically meaningful risk categories using only three features: tumor size, tumor grade and microvascular invasion, and a representative nomogram and a visual prediction surface were constructed using these features in Cox proportional hazards model. Concordance indices in the training and validation cohorts were 0.755 ± 0.029 and 0.836 ± 0.015 for our novel model, which were comparable to the C-indices of the original Leibovich prediction model (0.734 ± 0.035 and 0.848 ± 0.017, respectively). Thus, the presented model retains high accuracy while requiring only three features that are routinely collected and widely available.

## Introduction

After surgery for localized renal cell carcinoma (RCC) approximately 20 to 30% of patients will develop disease recurrence^1,2^ and a 5-year disease-free survival ranges from 91 to 51% in contemporary studies^3,4^. Tumor stage, higher nuclear grade, the presence of histologic tumor necrosis, microvascular invasion, sarcomatoid differentiation and positive surgical margins are associated with shorter disease-free and cancer-specific survival after radical nephrectomy (RN) and partial nephrectomy (PN)^3–8^. Risk-based follow-up with thoracic and abdominal computed tomography (CT) is recommended to detect distant metastases and local recurrence early^9,10^.

Several prediction models have been developed to assess oncologic outcomes after RN or PN (Table 1). The Kattan^2^, UISS^11^ and Karakiewicz^12^ prediction models include clear cell, papillary and chromophobe RCC, whereas the SSIGN^13^, Leibovich^8^ and Sorbellini^14^ models were developed for clear cell RCC only. In 2018 Leibovich et al. introduced new models to predict progression-free and cancer-specific survival in clear cell, papillary and chromophobe RCC separately^15^. To stratify patients into risk groups Kattan, Sorbellini and Karakiewicz presented their models as nomograms, UISS introduced a categorization table and SSIGN and Leibovich models were presented with a scoring table. The required features for the prediction task in different models are described in Table 1.

**Table 1:** Renal cell carcinoma postoperative prediction models.

Model	Outcome	C-index	Features
Kattan (2001)	RFS^a^	0.74	Symptoms (incidental, local, systemic symptoms), Histology (chromophobe, papillary, clear cell), Tumor size, 1997 pT-stage^2^
UISS (2001)	OS^b^	ND^e^	1997 TNM Stage, Fuhrman grade, ECOG performance status^11^
SSIGN (2002)	CSS^c^	0.84	1997 T stage, N stage, M stage, Tumor size, Fuhrman grade, Necrosis^13^
Leibovich (2003)	MFS^d^	0.819	Tumor Stage, Regional lymph node status, Tumor Size, Fuhrman grade, Necrosis^8^
Sorbellini (2005)	RFS	0.82	Size, 2002pT, Fuhrman grade, Necrosis, Vascular invasion, Presentation (incidental, local symptoms, systemic symptoms)^14^
Karakiewicz (2009)	CSS	ND	Age, Gender, Symptoms (no, local, systemic), Tumor Size, T-stage, Metastasis^12^
Leibovich (2018)	RFS	0.83	Constitutional symptoms (yes, no), WHO/ISUP 2016 tumor grade, Necrosis, Sarcomatoid differentiation, Tumor size, Perinephric or renal sinus fat invasion, Tumor thrombus level, Extension beyond kidney, Nodal involvement^15^

^a^Recurrence-free survival.

^b^Overall survival.

^c^Cancer-specific survival.

^d^Metastasis-free survival.

^e^Not defined.

In this study, our primary objective was to build an easy-to-use and accurate prognostic model using readily available clinicopathologic features to predict the risk of developing distant metastases after surgery of localized clear cell RCC. Focus was placed on selecting a minimal set of features that would still optimally predict recurrence. We further aimed to build a visual prediction surface to stratify patients into relevant risk groups for convenient adoption to clinical use. Patients with non-clear cell histology (e.g. papillary and chromophobe RCC) were excluded from our analysis as well as patients with local recurrences in the kidney after PN or in the retroperitoneal space after RN without distant metastases. Primarily metastatic patients who underwent cytoreductive nephrectomy were also excluded. The original Leibovich model^8^ was chosen as the reference model, because it has been thoroughly validated by other researchers^16,17^.

## Patients and methods

### Patient cohorts and features included in the analysis

A training cohort of 196 patients operated between 2005 and 2014 for localized clear cell RCC was identified from the pathology reports of Turku University Hospital, Finland. Clinical and pathological features and the outcome of the patients were retrospectively collected from original pathology and medical reports. The pathological features of primary tumor included the histologic subtype, tumor size (maximum dimension), TNM stage (according to AJCC 7th edition) and tumor grade as well as information on the presence of microvascular invasion, sarcomatoid differentiation, histologic tumor necrosis, a positive surgical margin and tumor invasion into the renal pelvis, ureter, peripelvic fat, perinephric fat, renal vein or ipsilateral adrenal gland. Microvascular invasion was defined as tumor cells within small vessels in the tumor pseudocapsule, tumor or renal parenchyma adjacent to the tumor^6^. No immunohistochemistry was used to detect microvascular invasion. In addition to the 4-tiered Fuhrman grading system^18^, the tumor grade was also determined using the 3-tiered WHO (1998) grading system^19^. Histological features were determined according to the initial pathology report and no centralized histological assessment was performed. Therefore, traditional Fuhrman grading was used instead of modern WHO/ISUP grading^6^ in our analysis. The clinical features included age, sex, the Charlson comorbidity index^20^, ASA classification, hemoglobin, hematocrit, the mean corpuscular volume, the mean corpuscular hemoglobin, white blood cells, platelets and serum creatinine at the time of surgery. A comprehensive list of all the tested prognostic features is reported in the Supplementary Table S1.

The primary endpoint was the clinical detection of distant metastases during postoperative follow-up with thoracic and abdominal CT. Biopsy was not required to confirm distant metastases. The follow-up cut-off date was 11.7.2019 for the training cohort. The Leibovich score was determined for each patient as described in the original article^8^.

The external validation cohort consisted of 714 patients, who underwent PN or RN for localized clear cell RCC at Helsinki University Hospital, Finland, between 2006 and 2015. Patients had postoperative follow-up with thoracic and abdominal CT according to local clinical practice to detect distant metastases. Biopsy was not required to confirm metastatic disease. Patients with non-clear cell histology, cytoreductive nephrectomy, and local recurrence without distant metastases were excluded. The follow-up cut-off date was 18.9.2019 for the validation cohort. After identifying the required predictive features from the training cohort, these features were gathered from the validation cohort together with the features required for calculating the Leibovich score. The Leibovich score was determined similarly as for the training cohort.

**Figure 1:**
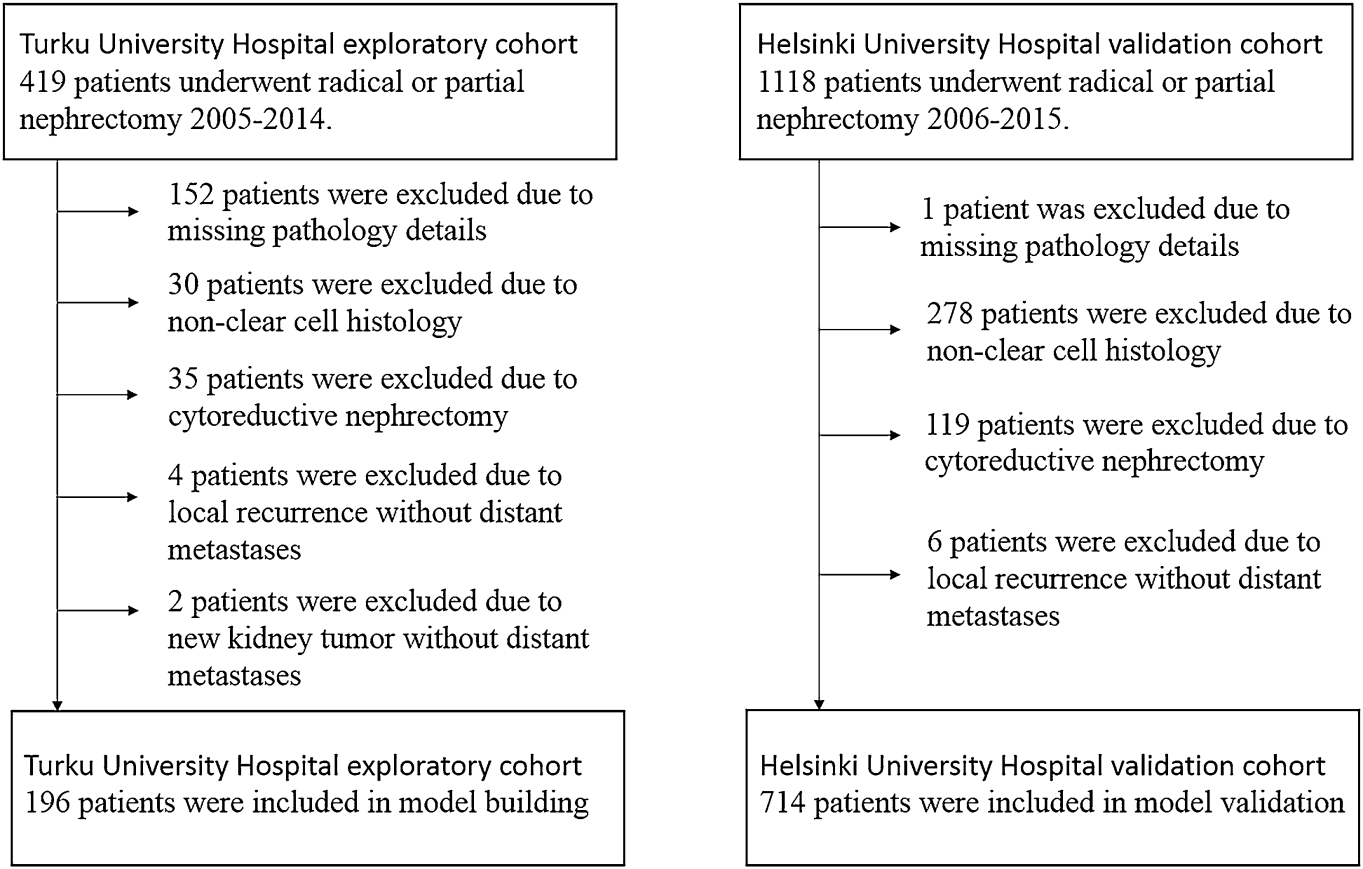
Patient selection flow chart.

Patient characteristics of the Turku University Hospital training cohort and the Helsinki University Hospital validation cohort are shown in Table 2. Patient selection for the Turku University Hospital training cohort and for the Helsinki University Hospital validation cohort is described in Fig. 1.

**Table 2:** Patient characteristics.

	Turku University Hospital training cohort (N = 196)	Helsinki University Hospital validation cohort (N = 714)
Age (years)^a^	67 (37–89)	66 (21–89)
Male/female^b^	118 (60%)/78 (40%)	393 (55%)/321 (45%)
**T stage**^**2**^		
1	104 (53%)	424 (59%)
2	32 (16%)	50 (7%)
3–4	59 (30%)	240 (33%)
Unknown	1 (< 1%)	–
**Regional nodal status**^**b**^		
Nx/N0	194 (99%)	703 (98%)
N1	2 (1%)	11 (2%)
Tumor size (mm)^a^	57 (10–160)	48 (8–200)
**Histologic tumor necrosis**^**b**^		
Yes	71 (36%)	148 (21%)
No	7 (4%)	563 (79%)
Unknown	118 (60%)	3 (< 1%)
**Microvascular invasion**^**b**^		
Yes	33 (17%)	127 (18%)
No	152 (78%)	587 (82%)
Unknown	11 (6%)	–
**Tumor grade (Fuhrman)**^**b**^		
1	32 (16%)	102 (14%)
2	87 (44%)	389 (55%)
3	60 (31%)	193 (27%)
4	17 (9%)	27 (4%)
Unknown	–	3 (< 1%)

Values reported as: ^a^Median (Range), ^b^Absolute amount (Percentage).

### Statistical modelling

During exploratory analyses, the Turku University Hospital training cohort was subjected to regularized Cox regression using the LASSO L1-norm for identifying optimal features in right-censored progression free survival. Tenfold cross-validation was run over 100 random binnings of the training cohort. At each of these 100 runs, a conservative lambda penalization parameter was selected at the first value that was within one standard error of the cross-validation error minimum. The features were then ordered according to how many times they appeared over the 100 runs, with the top three occurring features being maximum tumor diameter, tumor grade, and microvascular invasion status. After testing for C-index in the validation cohort in comparison with the Leibovich score, the final proposed Cox model was fit using the predetermined features for observations from both cohorts in order to leverage the larger N available in the validation cohort.

R statistical software v3.5.2 was utilized for all the statistical analyses. The R-packages glmnet, survival, survminer, compareC, rms, survivalROC and hamlet were utilized during model fitting, feature extraction, benchmarking and subsequent visualization steps for the nomogram and risk grouping surface. In the results section, continuous variables are presented with median and interquartile range and categorical variables with category amounts and corresponding percentages.

### Ethical approval statement

This study was approved by the institutional research board of Turku and Helsinki University Hospitals. Informed consent was waived due to retrospective nature of this study and was approved by IRB and ethics committee (Turku Clinical Research Centre Licence number T06/032/15, date 28.9.2015). All methods were performed in accordance with the institutional guidelines and regulations. Data was anonymized for statistical analyses and handled in a manner that meets the EU General Data Protection Regulation 2016/679 (GDPR) on data protection.

## Results

### Clinical outcome

The median follow-up in our RCC patient cohorts was 76.1 (interquartile range 40.9–103.9) months in the training cohort and 65.4 (47.7–90.8) months in the validation cohort. 55 (28%) patients in the training cohort and 134 (19%) patients in the validation cohort developed distant metastases during postoperative follow-up. The median time to distant metastases was 25.5 (11.2–49.9) months in the training cohort and 21.9 (8.8–42.8) months in the validation cohort. The most common sites of metastases were lungs (47% of the patients with metastases), lymph nodes (36%) and brain (16%) in the training cohort and lungs (68%), bone (16%) and lymph nodes (12%) in the validation cohort.

### Prognostic model for postoperative RCC outcome

We employed regularized Cox regression using the LASSO L1-norm to discover a minimal set of clinicopathologic features for predicting RCC metastases after surgery. Only three features, tumor size, tumor grade (Fuhrman) and microvascular invasion, were found to be essential for an accurate prediction model in the Turku University Hospital training cohort of 196 patients. We will henceforth refer to our prognostic modelling framework, i.e. building a statistical model for predicting a metastasis-free outcome for a patient, as a prediction model for convenience. The resulting model was then validated in the external Helsinki University Hospital cohort of 714 patients.

**Figure 2:**
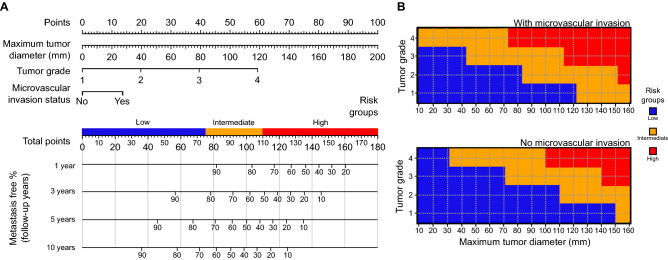
(**A**) A nomogram for calculating continuous risk scores for stratifying patients into low-, intermediate- and high-risk groups using the three identified features, presented together with 1-, 3-, 5- and 10-year metastasis free proportions associated with the total score. (**B**) The three-feature prediction surface for stratifying patients into the low-, intermediate- and high-risk groups.

**Figure 3:**
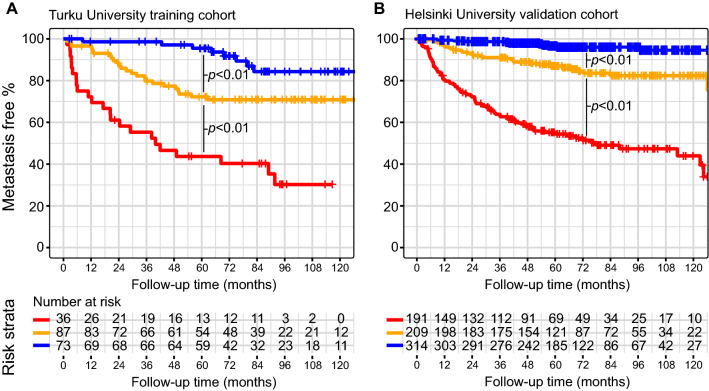
Kaplan–Meier curves illustrating statistically significant differences in metastasis-free survival in the training cohort (**A**) and in the validation cohort (**B**).

We stratified patients into three clinically relevant risk groups (low-, intermediate- and high-risk) in both cohorts using the proposed three-feature prediction model (Fig. 2). The metastasis-free survival of these risk groups is illustrated with Kaplan–Meier curves in Fig. 3. The final proposed Cox model is displayed in Table 3. Using calibration plots as diagnostics, we observed that the predicted risks from the Cox model agreed with the general trends for observed probabilities (Supplementary Fig. S1).

**Table 3:** The final Cox regression model obtained after feature extraction.

Feature	Levels	Parameter estimate	p-value	Hazard ratio* [95% CI]
Tumor max diameter	Increment in millimetres	0.017326	< 0.0001	1.017 [1.014–1.021]
Tumor grade (Fuhrman)	Increment in levels from 1 to 4	0.685226	< 0.0001	1.984 [1.604–2.454]
Microvascular invasion status	Positive finding reported	0.236717	0.0034	1.267 [1.081–1.485]

*CI* confidence interval.

*Ratio increment in hazard of event happening per level of feature when all other features are held constant.

In the training cohort 73 (37%), 87 (44%) and 36 (18%) patients were classified into the low-, intermediate- and high-risk groups, respectively. In the validation cohort 314 (44%), 209 (29%) and 191 (27%) patients were classified into the low-, intermediate- and high-risk groups. During postoperative follow-up, distant metastases were found in 8 (11%), 24 (28%) and 23 (64%) patients in the low-, intermediate- and high-risk group of the training cohort and in 12 (4%), 31 (15%) and 91 (48%) patients in the low-, intermediate- and high-risk group of the validation cohort. The median time to distant metastases in the low-, intermediate- and high-risk group was 66.6 (52.5–75.9) months, 26.0 (18.2–39.8) months and 17.5 (4.7–39.0) months in the training cohort and 46.6 (19.7–57.9) months, 24.2 (13.0–50.9) months and 18.5 (7.2–36.0) months in the validation cohort respectively. Low-risk patients developed metastases later compared to the intermediate- and high-risk patients indicating more indolent course of the disease.

**Figure 4:**
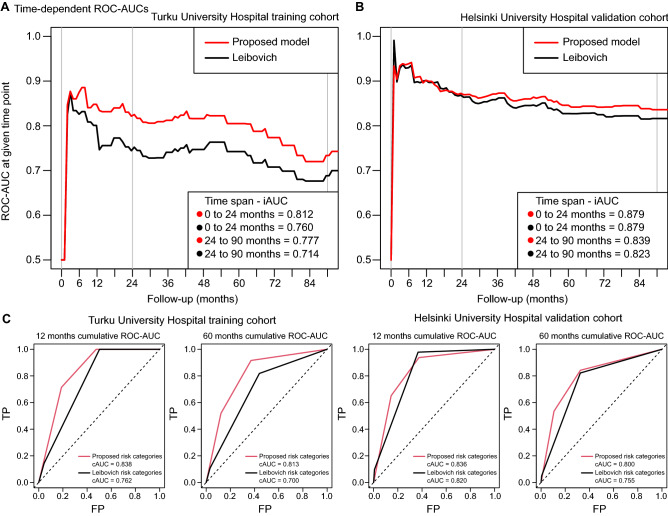
Time-dependent ROC-AUC performance for the proposed new model and the benchmarking Leibovich model in the training cohort (**A**) and the validation cohort (**B**). Integrated AUC (iAUC) was computed as the proportion of true performance in area under curve (AUC) within a given time interval out of a perfect performance (AUC at 1.0). Subsequently, both the proposed new model and the Leibovich model were stratified into three risk groups (low, intermediate, and high; (**C**)), and two representative time points at 1-year and 5-year follow-up are shown here for discriminative capability for both models using cumulative ROC-AUC.

The novel proposed model achieved high prediction accuracy. The C-index and standard error was 0.755 ± 0.029 in the training cohort and 0.836 ± 0.015 in the validation cohort. We then benchmarked our prediction model against the Leibovich model^8^. While our model outperformed the Leibovich model in the training cohort (C-index 0.734 ± 0.035), there was no statistically significant difference between our proposed prediction model and the Leibovich model (C-index 0.848 ± 0.017) in the validation cohort (p = 0.106). Noticeably however, the number of identified prediction features was lower in our model compared to the models introduced by Leibovich et al.^8,15^. The sensitivity and specificity of our proposed model and the Leibovich model is illustrated by time-dependent ROC-AUC-curves in Fig. 4. Noticeably, our novel model retained higher predictive accuracy in the later time points (24 to 90 months, Fig. 4A,B), suggesting that the smaller set of prognostic features was more robust for long-term predictions. Furthermore, we examined whether discretization of both risk scores into three risk categories would affect performance of either model; as observed in Fig. 4C, both models retained similar trends measured using cumulative ROC-AUC as their continuous counterparts presented in Fig. 4 panels A and B with time-dependent ROC-AUC.

### Visual prediction surface for postoperative RCC outcome

We developed a nomogram to calculate patient’s risk score using the three proposed features (Fig. 2A). Tumor size, tumor grade (Fuhrman) and microvascular invasion each result in points from 0 to 100 (tumor size), 0 to 60 (tumor grade) and 0 to 15 (microvascular invasion) and the total points from 0 to 180. Individual risk score is obtained by summing each separate risk factor. Furthermore, average metastasis-free survival rates are reported at 1, 3, 5 and 10 years of follow-up as associated with the sum of the risk score. As an alternative method, a visual prediction surface for determining the risk group given all three variables is presented in Fig. 2B. Low-risk patients are indicated by the blue colour, intermediate-risk patients by the yellow colour and high-risk patients by the red colour.

## Discussion

In this study we aimed to develop an easy-to-use prognostic model to assess metastasis-free survival after surgery of localized clear cell RCC. We introduced a model that required only three features, tumor size, tumor grade and microvascular invasion, for the prediction task. Tumor size was found to have the largest effect on metastases-free survival in our study. Our model confirmed that routinely reported microvascular invasion, even when not based on a centralized review or immunohistochemical staining, increases the risk of disease recurrence as noted in earlier studies^21,22^.

The Concordance index of our proposed model reached 0.836 in the validation cohort and was similar to the C-index of 0.848 for the original Leibovich model in the validation cohort (p = 0.106). The reported C-indices for other prediction models do not exceed 0.83^22^. The prediction accuracy of our model was also comparable to reported C-index of 0.83 for recurrence-free survival in the 2018 updated Leibovich model^15^. Interestingly, as shown in Fig. 4, our novel model has better prognostic value for long term prediction in both cohorts, as it retained a higher integrated AUC for the time-dependent ROC-AUC post 24 months. The original Leibovich model required five features (T-stage, N-stage, Tumor size, Nuclear grade and Histologic tumor necrosis)^8^ and the updated Leibovich model required nine features (Constitutional symptoms, Tumor grade, Coagulative necrosis, Sarcomatoid differentiation, Tumor size, Perinephric or renal sinus fat invasion, Tumor thrombus level, Extension beyond kidney and Nodal involvement)^15^ for prediction task, whereas our proposed model reached comparable prediction accuracy with only three readily available features of the primary tumor. Because of the lack of the information on constitutional symptoms and tumor thrombus level in our study cohorts, we were not able to compare our model directly with the updated Leibovich model.

The limitations of our study include its retrospective nature, the small number (196 patients) of patients in the training cohort and the lack of centralized re-review of the original pathology reports However, this was compensated by the size of the validation cohort (714 patients) and by fitting the final Cox model utilizing patients from both cohorts to obtain more reliable estimates. Pathologic features were collected from the original pathology reports and therefore Fuhrman grading was used instead of WHO/ISUP grading. The information on tumor necrosis was missing in 60% of the original pathology reports of the Turku University Hospital cohort but it was routinely reported in the Helsinki University Hospital cohort (< 1% missing). Because of the retrospective nature of our study, there was no standardized follow-up protocol and postoperative imaging was performed according to local clinical practice at Turku and Helsinki University Hospitals. The proportion of patients that underwent PN increased from 8% in 2007 to 23% in 2014 in the Turku University Hospital cohort, but the surgical technique has not been observed to affect the risk of distant metastases^23^. However, we decided to exclude patients with local recurrence in the kidney after PN and in the retroperitoneal space after RN without distant metastases in order to eliminate the effect of surgical technique on our analysis. Importantly, the simple yet efficient prognostic model was successfully validated in the larger external validation cohort. A systematic, prospective evaluation of our model with other prognostic models in a larger patient population is warranted to support wider clinical use of our model, as retrospective prognostic models have been found to overestimate their accuracy in prospective settings^24^. Furthermore, our training data may have underrepresented otherwise informative clinical factors, such as necrosis, and may therefore present bias towards features that were widely available in the training cohort.

In conclusion, only three tumour features (tumor size, tumor grade and microvascular invasion) were sufficient for the prediction of metastasis-free survival after surgery of localized clear cell RCC with a similar prediction accuracy as the 2003 Leibovich model. To facilitate the use of our model, a nomogram (Fig. 2A) and a risk grouping surface (Fig. 2B) were introduced to assess each patient’s relative risk scores and to subsequently stratify patients into clinically relevant risk groups.

## Supplementary Information

Supplementary Information.

## Data Availability

Data is available upon request from the corresponding author.
