# Supplementary material for: A three-feature prediction model for metastasis-free survival after surgery of localized clear cell renal cell carcinoma
Source: Sci Rep. 2021 Apr 21;11:8650. doi: 10.1038/s41598-021-88177-9 (PMC8060273; doi:10.1038/s41598-021-88177-9)
Supplement: Supplementary file 1 — Supplementary Information. [file 41598_2021_88177_MOESM1_ESM.doc]

A Three-feature Prediction Model for Metastasis-free Survival after Surgery of Localized Clear Cell Renal Cell Carcinoma

Kalle E. Mattila^a^*, Teemu D. Laajala^bc^*, Sara V. Tornberg^d^, Tuomas P. Kilpeläinen^d^, Paula Vainio^e^, Otto Ettala^f^, Peter J. Boström^f^, Harry Nisen^d^, Laura L. Elo^c+^, Panu M. Jaakkola^a,c+^

^a^Fican West Cancer Centre, Department of Oncology and Radiotherapy, University of Turku and Turku University Hospital, Turku, Finland; ^b^Fican West Cancer Centre, Biomathematics Research Group, University of Turku, Turku, Finland; ^c^Turku Bioscience Centre, University of Turku and Åbo Akademi University, Turku, Finland; ^d^Department of Urology, University of Helsinki and Helsinki University Hospital, Helsinki, Finland; ^e^Department of Pathology, University of Turku and Turku University Hospital, Turku, Finland;  ^f^Department of Urology, University of Turku and Turku University Hospital Turku, Finland.

*equal contribution (first authors)

^+^equal contribution (senior authors)

*Kalle E Mattila, M.D., Fican West Cancer Centre, Department of Oncology and Radiotherapy, Turku University Hospital, Hämeentie 11, Post Box 52, FIN-20521, Turku, Finland. Tel: +358-2-3130729, Fax: +358-2-3132809, [kalle.mattila@tyks.fi](mailto:kalle.mattila@tyks.fi)

### Supplementary Table S1. List of all original or derived variables subjected to feature selection for the disease progression prediction. The columns indicate a general short variable name, variable type or properties (e.g. a positive numeric lab measurement or a binary indicator for 0=NO, 1=YES), if the variable was identified as important and included in the final predictive model, and a more extensive description of the variable.

| Variable | Type | Included | Description |
| --- | --- | --- | --- |
| Age | Integer |  | Patient’s age in years |
| Gender | Binary |  | Binary indicator for Male/Female |
| ASA classification | Integer |  | ASA physical status classification system |
| Tumor size | Integer | x | Positive measurement unit in millimeters (maximum dimension) |
| WHO grade | Integer |  | Tumor grading 1/2/3 WHO 1998 |
| Fuhrman grade | Integer | x | Tumor grading 1/2/3/4 |
| Charlson comobidity index | Integer |  | Charlson comorbidity index |
| Peripelvic fat infiltration | Binary |  | Tumor invasion to peripelvic fat |
| Perirenal fat infiltration | Binary |  | Tumor invasion to perirenal fat |
| Growth to renal pelvis | Binary |  | Tumor invasion to renal pelvis |
| Positive surgical margins | Binary |  | Tumor invasion to surgical margins |
| Growth to ureter | Binary |  | Tumor invasion to ureter |
| Microscopic vessel invasion | Binary | x | Tumor cells within small vessels in the tumor pseudo-capsule, tumor or renal parenchyma adjacent to the tumor |
| Macroscopic vessel invasion | Binary |  | Tumor invasion to renal vein |
| Growth to adrenal gland | Binary |  | Tumor invasion to adrenal gland |
| Clear cell carcinoma or other | Binary |  | Binary indicator if histology was determined as CCC or non-CCC |
| T classification | Integer |  | Stepwise testing of incrementing T classification (TNM) |
| Nx / N0 / N1 / N2+ | Integer |  | Metastatic status of examined lymph nodes |
| Hemoglobin | Positive numeric |  | Laboratory measurement; average, lowest and highest measurements prior to surgery |
| Hematocrite | Positive numeric |  | Laboratory measurement; average, lowest and highest measurements prior to surgery |
| Erythrocytes | Positive numeric |  | Laboratory measurement; average, lowest and highest measurements prior to surgery |
| The mean corpuscular hemoglobin | Positive numeric |  | Laboratory measurement; average, lowest and highest measurements prior to surgery |
| The mean corpuscular volume | Positive numeric |  | Laboratory measurement; average, lowest and highest measurements prior to surgery |
| Leukocytes | Positive numeric |  | Laboratory measurement; average, lowest and highest measurements prior to surgery |
| Thrombocytes | Positive numeric |  | Laboratory measurement; average, lowest and highest measurements prior to surgery |
| Creatinine | Positive numeric |  | Laboratory measurement; average, lowest and highest measurements prior to surgery |
| Hard-thresholds on continuous variables | Binary |  | E.g. tumor largest dimension ≥ 10 cm binary indicator and similarly in a grid for other continuous variables. |
| WHO grade thresholds | Binary |  | WHO grade ≥ 1 or 2 as a binary threshold. |
| Fuhrman grade thresholds | Binary |  | Fuhrman grade ≥ 1, 2 or 3 as a binary threshold. |
| Charlson index thresholds | Binary |  | Charlson comorbidity index ≥ 2, 4, 6, 8, or 10 as a binary threshold. |

Supplementary Figure S1. Model calibration plot in the two cohorts. Overall, while slight discrepancies existed for some risk prediction quantiles, the predicted risks followed the trends observed in the data.
